# CapBuild: a cloud-native tool for adeno-associated virus capsid engineering

**DOI:** 10.1093/nar/gkaf422

**Published:** 2025-05-16

**Authors:** Anne H Klein, Michael J Kuiper, Mark Burgess, Anuradha Wickramarachchi, Yatish Jain, Denis C Bauer, Laurence Wilson

**Affiliations:** Australian e-Health Research Centre, Commonwealth Scientific and Industrial Research Organisation (CSIRO), Herston, Queensland 4006, Australia; Data 61, Commonwealth Scientific and Industrial Research Organisation, Canberra, Australian Capital Territory 2601, Australia; Australian e-Health Research Centre, Commonwealth Scientific and Industrial Research Organisation (CSIRO), Canberra, Australian Capital Territory 2601, Australia; Australian e-Health Research Centre, Commonwealth Scientific and Industrial Research Organisation (CSIRO), Adelaide, South Australia 5064, Australia; Australian e-Health Research Centre, Commonwealth Scientific and Industrial Research Organisation (CSIRO), Westmead, Sydney 2145, Australia; Department of Applied BioSciences, Faculty of Science and Engineering, Macquarie University, Macquarie Park, New South Wales 2113, Australia; Department of Applied BioSciences, Faculty of Science and Engineering, Macquarie University, Macquarie Park, New South Wales 2113, Australia; Department of Biomedical Sciences, Macquarie University, Macquarie Park, New South Wales 2113, Australia; Australian e-Health Research Centre, Commonwealth Scientific and Industrial Research Organisation (CSIRO), Adelaide, South Australia 5064, Australia; Department of Applied BioSciences, Faculty of Science and Engineering, Macquarie University, Macquarie Park, New South Wales 2113, Australia; Australian e-Health Research Centre, Commonwealth Scientific and Industrial Research Organisation (CSIRO), Westmead, Sydney 2145, Australia

## Abstract

Adeno-associated virus (AAV) capsid engineering is essential for advancing gene therapy but remains limited by structural complexity and computational constraints. To address these challenges, we developed CapBuild, a cloud-native web server that streamlines AAV capsid prediction, assembly and engineering. CapBuild provides two distinct workflows: a PDB-based pipeline for assembling complete capsids from structural files and a modelling pipeline that constructs capsids from protein sequences via SWISS-MODEL. The platform incorporates icosahedral symmetry through transformation matrices and features an integrated mutation modeller for visualising site-specific mutations across the entire capsid. Additionally, its amino acid localisation tool maps exposed and buried residues, facilitating rational design. Benchmarking against crystal structures demonstrates high structural accuracy, with consistently low RMSD values (0–0.89 Å) and high GDT scores (89.4–100%) across multiple AAV serotypes. CapBuild’s interactive visualisation interface, powered by Mol*, enables in-depth structural analysis, making capsid engineering more accessible to researchers. By reducing technical barriers and automating complex modelling tasks, CapBuild facilitates early-stage AAV capsid design, enabling researchers to rationally explore and visualise structural variants for potential use. CapBuild is available at https://capbuild.csiro.au/.

## Graphical Abstract

**Graphical Abstract:**
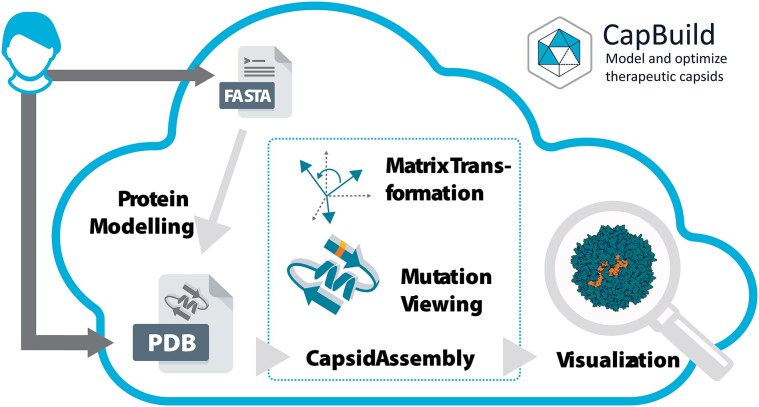

## Introduction

Adeno-associated virus (AAV) is the most widely used vector for gene therapy and is involved in numerous ongoing clinical trials in the field [1]. This popularity is attributed to their tissue tropism, low immunogenicity, absence of incorporation into the host chromosome, and long-lasting delivered gene expression [2–5]. The field of gene therapy has been transformed by potential of these vectors. This progress has facilitated the creation of variants exhibiting superior tissue specificity, diminished immunogenicity and enhanced transduction efficiency [3,6, 7].

Designing and optimising AAV capsids however, remains a significant challenge due to their intricate structure. The capsid shell consists of 60 copies of three Viral Protein (VP) sub-units (VP1, VP2 and VP3), existing in a specific ratio and arranged in strict symmetry [8, 9]. Developing new or iterating on existing capsids means understanding how changes to the amino-acid sequence of one subunit impacts the entire complex structure [10, 11]. Traditionally, capsid structure prediction and assembly have relied on experimental techniques such as X-ray crystallography and cryo-electron microscopy [12, 13]. While these techniques yield high-resolution structural data, they are characterised by significant temporal and financial investments and are frequently beyond the reach of researchers who lack access to specialised equipment. Computational approaches such as AlphaFold, which provides highly accurate structure predictions, homology modelling, machine learning-guided direct evolution and molecular dynamics simulations, have emerged as powerful alternatives, but existing tools often require substantial expertise or are constrained in their scope [14–16]. Notably, software such as CapsidBuilder (https://github.com/KCL-iGEM/Capacity-2019-) and CAPLIB [17] allow for the modelling and optimisation of capsid structures but require local installation and computational expertise. Meanwhile, VIPERdb [18] provides a web-based approach for capsid modelling but is limited to predefined structural conventions and lacks real-time visualisation or interactive mutation modelling. These limitations highlight the need for a more accessible, interactive and streamlined solution that facilitates the assembly of complete viral capsids and enables systematic mutagenesis at a capsid-wide level.

To address this gap, we present CapBuild, a cloud-native web server designed for AAV capsid prediction, assembly, and engineering. CapBuild distinguishes itself from existing computational tools not only through its accessibility but also by offering a suite of unique features that facilitate the capsid design process. CapBuild provides distinct pipelines tailored to different user requirements, allowing researchers to construct full capsids from either PDB files or protein sequences via SWISS-MODEL [19]. These capabilities, combined with automated symmetry generation using transformation matrices and interactive visualisation powered by Mol* [20], render CapBuild a comprehensive platform for rational AAV capsid design.

## Material and methods

### CapBuild method overview

CapBuild enables the modelling of full AAV capsids through two pathways: a PDB-based modelling pipeline and a sequence-based modelling pipeline. The PDB-based pipeline enables users to upload their own structural files for the different VP components in PDB format. Conversely, the sequence-based pipeline enables users to predict capsid structure from just the amino-acid sequences of a subunit, predicting its structure using a default PDB template and modelling the resulting capsid. Both pipelines make use of a suite of protein-modelling tools, integrated in a streamlined and user-friendly interface, as Mol* serves as the primary visualisation platform for exploring structural models, analysing molecular interactions and assessing the effects of mutations in real time.

### Modelling capsid structure from user PDB files

The PDB pipeline begins by evaluating the quality of the PDB file using pdb-tools to ensure compatibility with subsequent processing steps [21]. User-provided PDB files are then aligned to a reference structure, specifically the VP3 subunit of AAV1 (RCSB ID: 6JCR), using the Superimposer class from the Biopython PDB module [22]. This process involves extracting alpha-carbon atoms, calculating a transformation matrix to minimise root-mean-square deviation (RMSD), and applying the transformation to achieve precise alignment with the template.

After the cleaning and alignment steps, the pipeline employs transformation matrices rooted in Caspar-Klug Theory to replicate the input VP subunits into 60 symmetrically arranged units, conforming to the natural icosahedral geometry of AAV capsids [23]. These transformation matrices are derived from the cryo-EM structure of AAV1 (RCSB ID: 6JCR).

To ensure accurate capsid assembly, our pipeline automatically detects and grafts missing residues. User-provided sequences are aligned back to a reference and then missing amino-acids grafted process uses a template structure predicted using AlphaFold2 to add the missing residues with proper peptide bond connections to the user’s input structure [1–7]. This template structure is derived from the default AAV2 VP3 sequence that we predicted using AlphaFold and is available for download from the CapBuild landing page. This process in particular assists with missing N-terminal residues of VP3 which are often unresolved and missing from Cryo-EM structures and ensures that capsids are assembled correctly.

If the user has enabled VP2 and VP1, they are required to specify a ratio for the three components that adds up to 60 subunits. Each VP subunit is randomly assigned to the capsid segments, ensuring the final assembly complies with the specified ratio while preserving the capsid's icosahedral symmetry.

**Figure 1.**
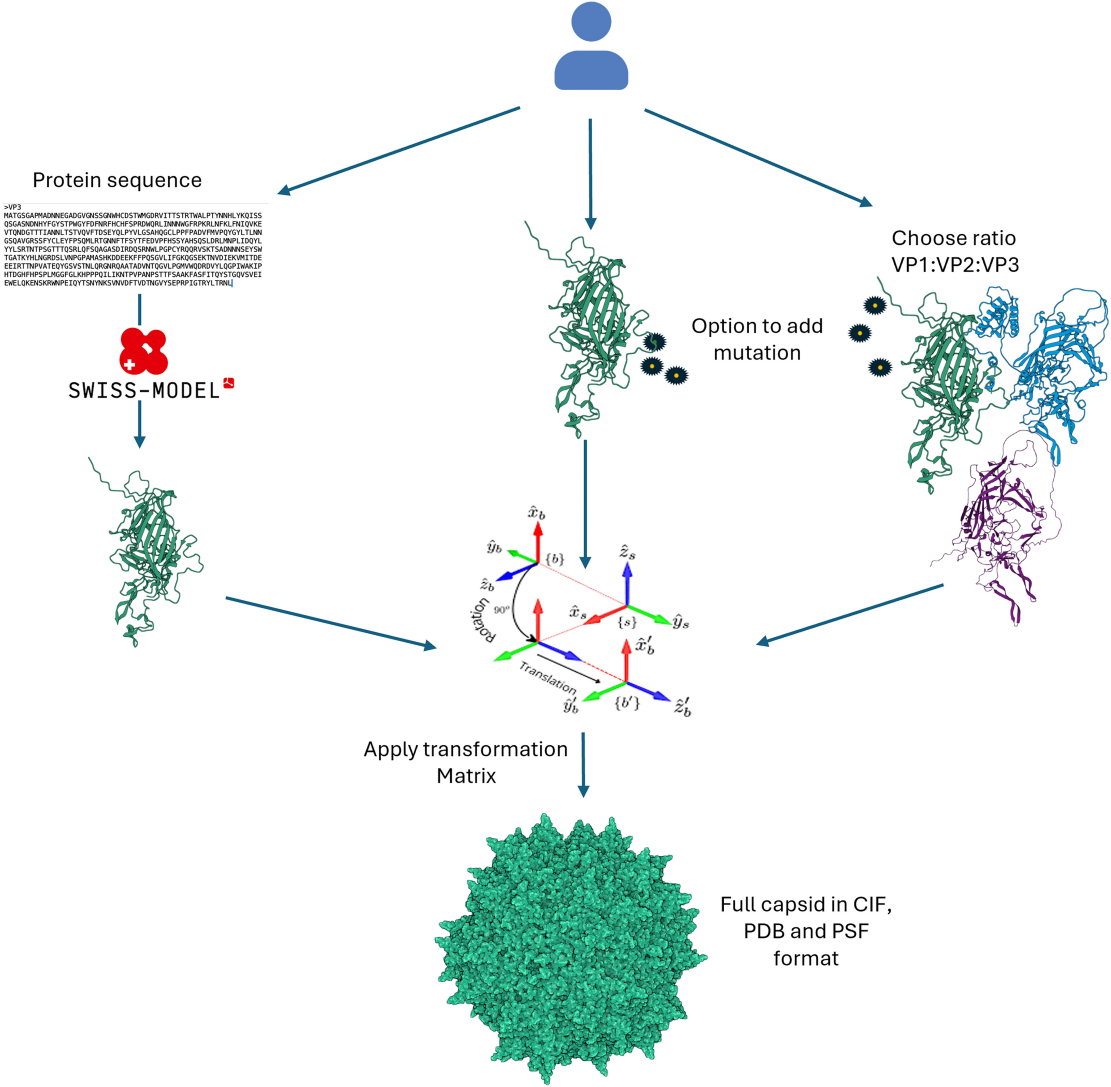
Overview of CapBuild’s PDB and modelling pipelines. The capsid assembly process can be initiated using either a protein sequence or structural data. When a protein sequence is provided (i), the structural model of the VP subunit is generated using SWISS-MODEL. Alternatively, a single PDB file of the VP3 subunit (ii) or three distinct PDB files corresponding to VP1, VP2 and VP3 (iii) can be used as input, with the latter allowing for a user-defined stoichiometric ratio of subunits. In both structural input approaches, site-specific mutations can be introduced if required. Regardless of the chosen pipeline, all approaches employ a transformation matrix to systematically position and orient the VP subunits, ensuring accurate icosahedral capsid assembly. The final capsid structure is exported in CIF, PDB and PSF formats.

The output of this step is a set of 60 individual PDB files, each corresponding to one subunit of the capsid that is subsequently assembled into the final capsid structure using a VMD custom script (Fig. 1). For the pipeline that includes VP1 and VP2 subunits, the system provides a warning about the lower structural certainty of these components, as their N-terminal regions are unresolved in experimental structures and their full structure is currently unknown. Ultimately, the assembled capsid structure is written into a Protein Structure File (PSF) in PDB and Crystallographic Information File (CIF) formats.

### Modelling capsid structure from amino-acid sequence

The modelling-pipeline is designed for users who do not have pre-assembled structural data and instead rely on FASTA sequences of the VP subunit. Within this workflow, the FASTA sequences are utilised to predict the three-dimensional structures of the subunits using the SWISS-MODEL ‘User Template’ mode. The predicted structures are subsequently assembled using the same methodology employed in the PDB Pipeline, including alignment, and replication using transformation matrices (Fig. 1).

### Advanced engineering features

The PDB pipeline incorporates optional features such as mutation modelling. Users can provide a list of specific amino-acid substitutions in the capsid subunit formats during the processing stage. Mutations are introduced by modifying the residue names in the PDB files, ensuring that the structural changes are reflected in the final capsid model. A log of the applied mutations is generated for the user reference.

Along with assembling the capsid, the pipeline can generate an amino acid map by assigning beta values to the PDB structure of the capsid. These values are calculated as the Euclidean distance (d) of each atom's coordinates (x, y, z) from the origin (0, 0, 0), using the following formula:


\begin{eqnarray*}
d\ = \ \sqrt {{{x}^2} + \ {{y}^2} + \ {{z}^2}}
\end{eqnarray*}

These distances are normalised and stored in the beta column of the PDB file, providing a spatial representation of each atom's relative position within the capsid. Additionally, the distances are compared to the average distance of all CA atoms in the structure. If the distance of a CA atom is greater than the average, its beta value is set to 1.00 and classified as ‘distal’, indicating the amino acid is located further from the centre of the capsid. Conversely, if the distance is less than or equal to the average, the beta value is set to −1.00 classified as ‘proximal’, signifying that the amino acid’s proximity to the inside of the capsid. This classification is subsequently saved in a CSV file for user reference.

### Webserver architecture

**Figure 2.**
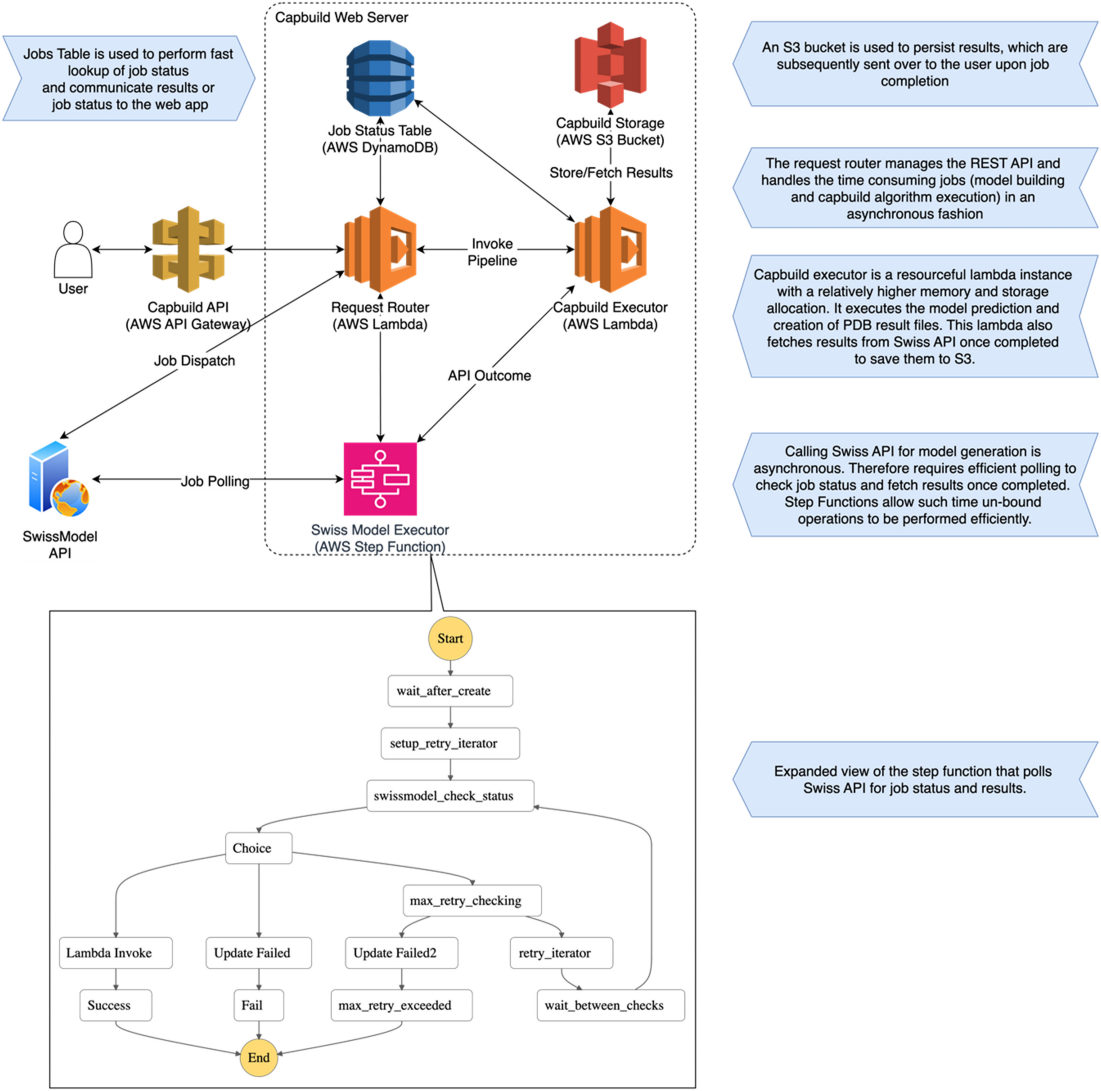
Architecture of the Capbuild Web Server. The webapp is distributed using AWS CloudFront service, which is a content delivery network (CDN) that offers high availability at a lower cost. The webapp automatically upload the files and sends other information from the users to dispatch the corresponding pipeline, Swiss Model API based approach or the PDB input approach. Particularly the PDB pipeline takes the uploaded PDB file and stores it in the AWS S3 bucket, thereafter performs coordinate matching with the VMD software to produce a matrix rotation producing PDB files of a complete capsid.

CapBuild is implemented using AWS serverless architecture, making it highly scalable and resource efficient. Fig. 2 illustrates the overall architecture of the Capbuild Web Server. User engagement is primarily facilitated through the Angular Webapp and subsequently the communication between the Webapp and the server is handled by the AWS Api Gateway. Requests to the server are using a Router Lambda function, which uses just 128MB of memory. This function routes the requests between the Swiss Model API and the Capbuild Lambda function. Swiss Model API is used for protein structure prediction, whereas the Capbuild Lambda performs matrix operations and the execution of the VMD Binary. Note that due to resource demanding nature of the Capbuild Lambda function, we have allocated 4GB of memory. The calls to Swiss Model API is handled via an AWS Step Function definition. The API performs in an asynchronous fashion where the initial request dispatches the job and additional API endpoints are provided to check the job status and fetch the results. Step function streamlines the process of polling and managing the state of the job without consuming valuable idle compute times. Once the Swiss Model API finishes (or fails) the Step Function triggers the CapBuild Lambda to carry out rest of the steps. Finally, the results are serialised as a collection of PDB, CIF and PSF files in the AWS S3 storage. We use DynamoDB to manage the state of the CapBuild Webserver jobs in a serverless manner.

We present the results of the CapbBuild Pipeline to users using the Mol* viewer. Additional files representing the logs, PDF, PDB and CIF formats are presented to the user for downloading.

The Modelling pipeline features extra steps where a sequence is uploaded and submitted to SwissModel API. Subsequently, the API is polled and once completed results are placed in the S3 bucket. Finally, they are fed into the same VMD for matrix rotation to produce a complete capsid.

### Protein structure files

All protein structures used in this paper were sourced from the RCSB Protein Data Bank. Structures were downloaded as PDB files using the provided RCSB IDs as identifiers.

## Results

### Benchmarking CapBuild’s pipelines

To assess the performance and accuracy of CapBuild’s pipelines, predicted structures were benchmarked against the cryo-EM structure of AAV1 (RCSB ID: 6JCR), a well-characterised AAV reference and used here for alignment and as the source of transformation matrices applied in the platform. Both the PDB pipeline and the modelling pipeline were evaluated.

**Figure 3.**
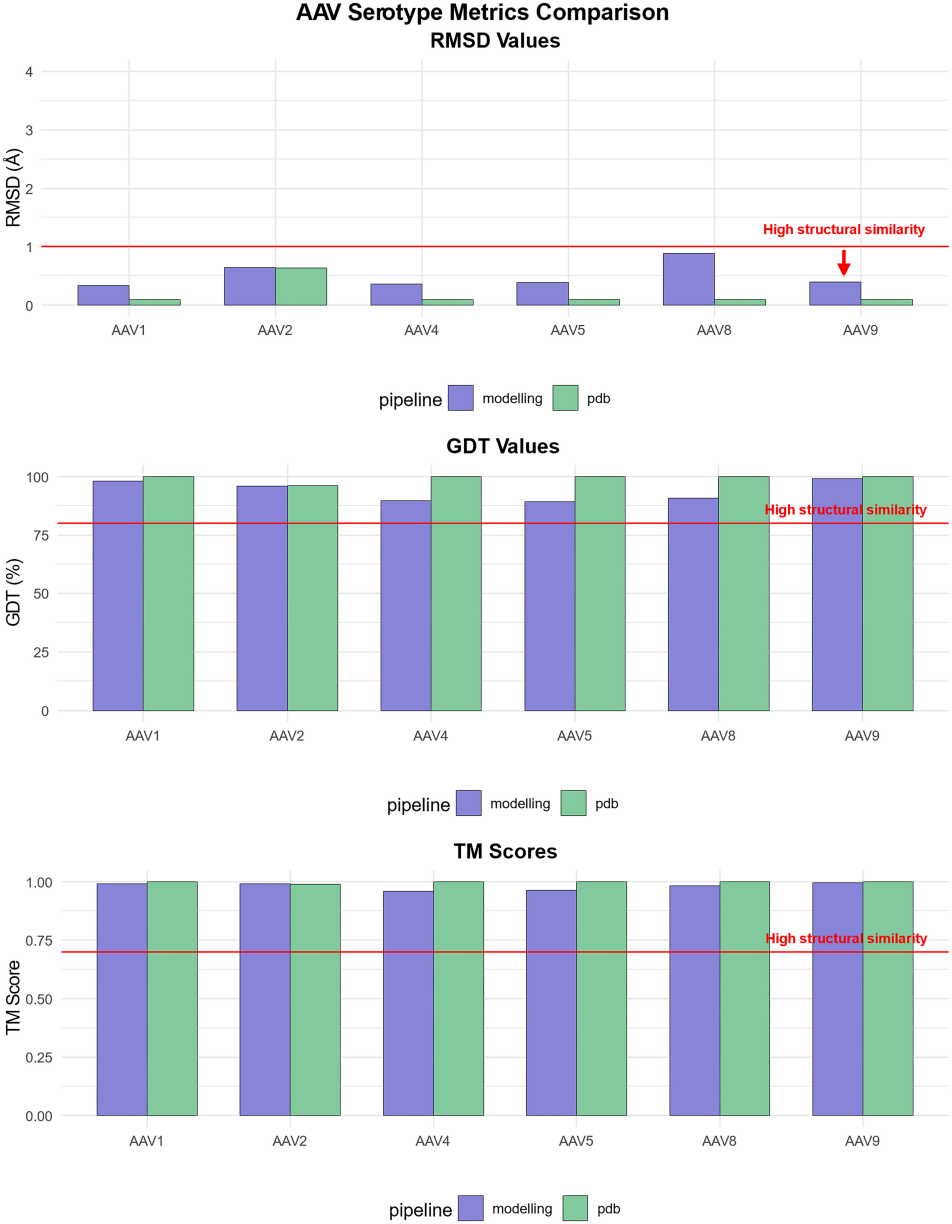
CapBuild predictions were benchmarked by predicting the structure of various AAVs and then comparing to experimentally determined structures using different metrics. CapBuild predictions showed low RMSD values (top), high GDT values (middle) and high TM scores (bottom), consistent with high structural similarity confirming that the CapBuild predictions are accurate.

**Figure 4.**
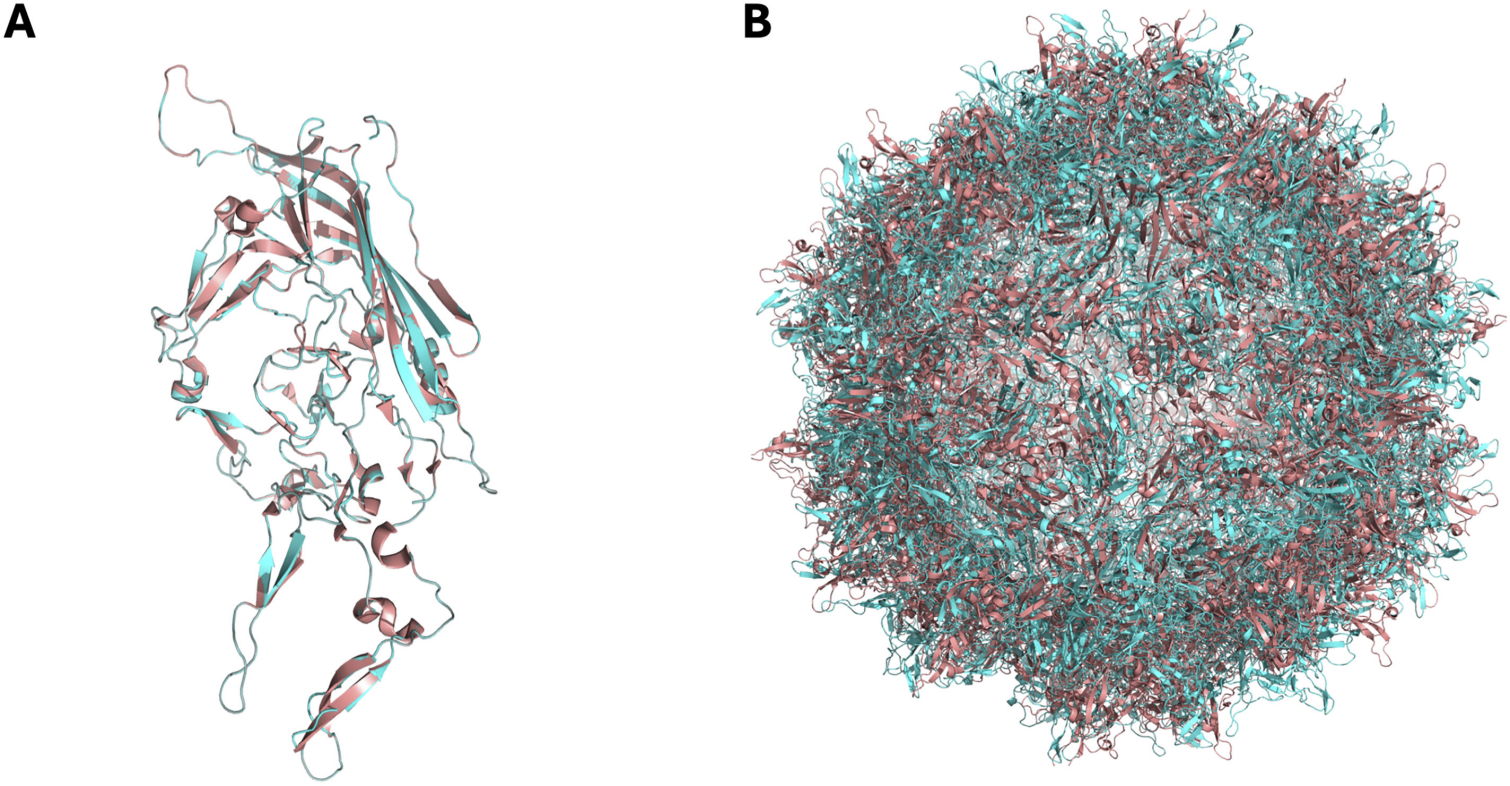
CapBuild structural predictions show high correlation with experimentally determined structure. (**A**) The predicted structure (red) of an AAV1 VP3 subunit was overlayed with the experimentally determined structure (blue, RCSB ID = 6JCR) showing high conformation. (**B**) This correlation was also observed at the full-capsid level with the predicted capsid structure overlapping with the experimentally determined structure.

Structural similarity was assessed using multiple metrics. RMSD measures the average distance between corresponding atoms in two structures, with lower values indicating higher similarity; an RMSD below 1 Å is typically considered a strong structural match [17]. The Global Distance Test (GDT) [24] measures the fraction of residues within a certain distance threshold between the compared structures, with values closer to 100% representing better alignment. A GDT score above 80% is generally considered to indicate high structural similarity [25]. Template modelling (TM) score [26] quantifies the overall structural similarity on a scale from 0 to 1, where scores above 0.5 generally indicate meaningful structural similarity, and scores above 0.7 suggest highly reliable structural alignment [27]. Each metric confirmed the structural fidelity of CapBuild's predictions, with low RMSD values and consistently high GDT and TM scores observed across models (Fig. 3). We extended the analysis to include comparisons to high-resolution structures of other serotypes (minimum resolution: 3.5–4 Å) including AAV2 (RCSB ID = 8FZ0), AAV4 (RCSB ID = 7THR), AAV5 (RCSB ID = 7KP3), AAV8 (RCSB ID = 6U2V) and AAV9 (RCSB ID = 7MTG). Using AAV1 as the reference template, the predicted capsid structures of these serotypes were generated using CapBuild and compared with their respective crystal structures, demonstrating robust performance across all cases. This high correlation is also observed when the CapBuild structure is overlaid with the experimentally determined structure (Fig. 4).

### Example use cases for CapBuild

Integration with Mol* allows for flexible and useful visualisation of the fully assembled capsid. To demonstrate the versatility and efficacy of CapBuild in addressing key challenges in AAV capsid engineering, we present three representative use cases that leverage the Mol* integration to illustrate the platform's capacity to model, analyse, and engineer capsids. Each example elucidates the platform’s practical applications focusing on leveraging its core functionalities for specific research objectives.

### Constructing a full capsid from a single subunit

**Figure 5.**
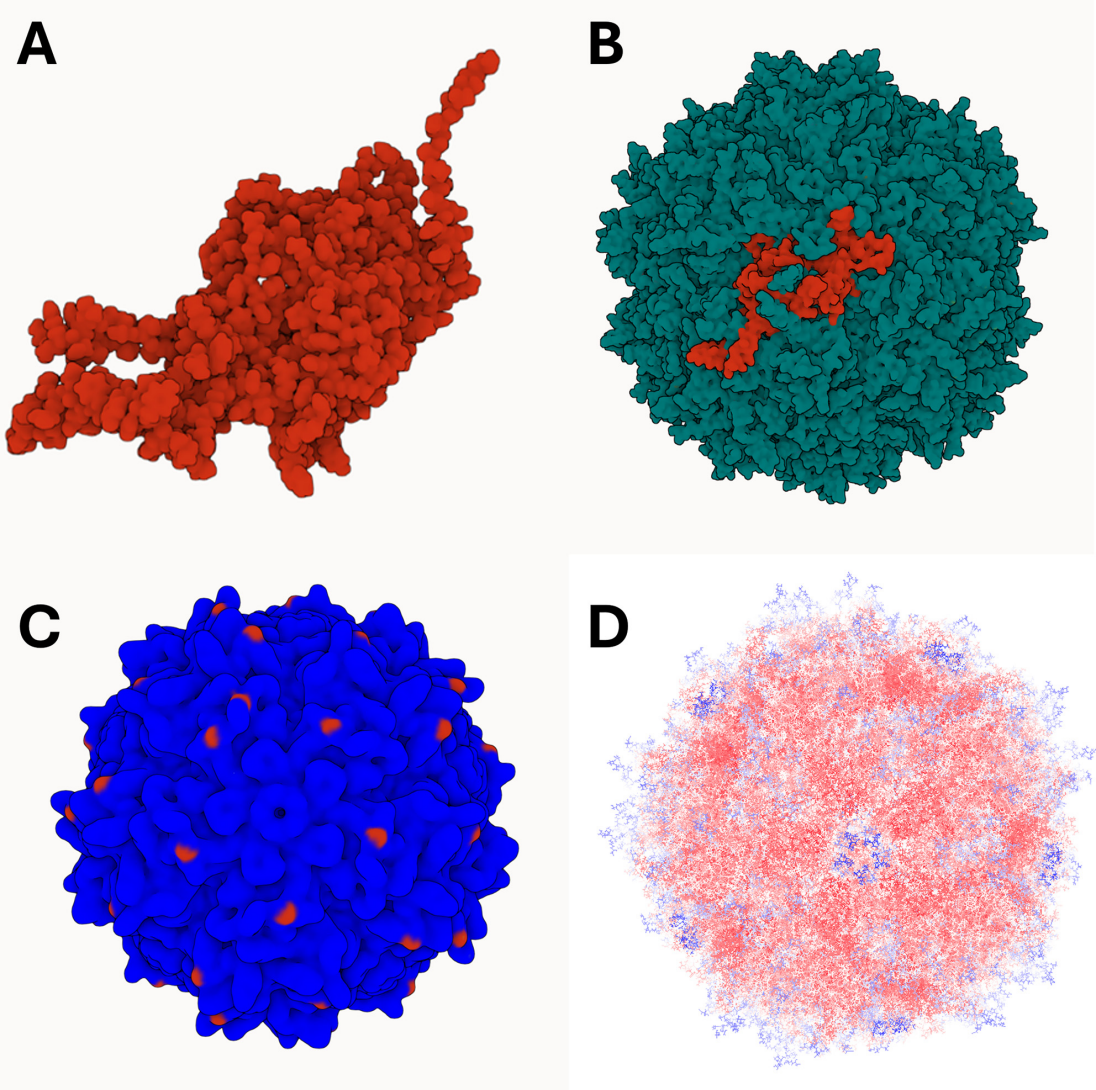
Example Outputs: An entire AAV2 capsid is generated from a single VP3 subunit. The AAV2 VP3 subunit can be visualised in isolation (**A**) or as part of an entire capsid (**B**). Selected amino-acids can be highlighted to visualise where they fall across the full capsid structure. This can be used to visualise where specific mutations are localised (**C**). A gradient-coloured AAV2 capsid visualised allowing the user to identify amino-acids that are distal from the central point (blue) and those that are proximal (red) (**D**).

Researchers often commence with only the structural information of a single AAV capsid subunit, available either as a PDB file or a FASTA sequence. Their objective is to reconstruct the entire capsid to investigate its structural characteristics, identify functional regions, or prepare the model for further engineering applications. This process, which traditionally requires advanced computational expertise and specialised tools, is significantly simplified with CapBuild. Through the PDB or modelling pipelines, CapBuild replicates the subunit symmetrically using transformation matrices, generating an accurate icosahedral capsid of 60 subunits. The assembled structure is rendered in the integrated Mol* viewer, enabling interactive exploration of the capsid. Fig. 5A and B demonstrates how an AAV2 subunit in PDB format was used to successfully construct and visualise the entire capsid using the PDB pipeline of CapBuild.

### Visualising mutations across a capsid structure

In numerous investigations, researchers endeavour to elucidate the influence of specific mutations on the structure and function of AAV capsids. CapBuild provides an advanced feature that enables users to introduce and visualise site-specific mutations symmetrically across the entire capsid. Fig. 5C demonstrates how a single mutation introduced in a VP3 subunit is propagated across the entire capsid using CapBuild, with the mutated residues distinctly highlighted for precise examination. This visualisation facilitates comprehensive inspection and comparative analyses, enabling researchers to assess local structural changes and their broader implications for capsid function.

### Mapping amino acid

A critical aspect of capsid engineering involves understanding the spatial distribution of residues to identify functional regions and evaluate structural accessibility. CapBuild addresses this need by generating amino acid localisation maps that differentiate between residues closer to the capsid centre (proximal residues) to those more distal. Through the calculation of Euclidean distances from the capsid centre, these spatial annotations are embedded within the beta column of the PDB file and are summarised in CSV file. As for Fig. 5D, the PDB annotated capsid structure can be visualised in molecular viewer like VMD with residues colour-coded to represent their positions, facilitating investigations into residue clustering and potential surface accessibility.

## Discussion

CapBuild provides a comprehensive and user-friendly platform for modelling and analysing AAV capsids with direct applications in rational variant design. offering early-stage insights that can assist in streamlining the development process. By offering two distinct pipelines the PDB pipeline for users with structural data and the modelling pipeline for those with only sequence information, CapBuild facilitates research for investigators with varying levels of computational expertise. Its ability to streamline the assembly of full icosahedral capsids and incorporate advanced engineering features, such as mutation modelling and amino acid localisation mapping, renders it a versatile tool for a wide range of applications in AAV research.

The platform has demonstrated robust performance and accuracy, as evidenced by benchmarking against cryo-EM structures of AAV1 and other serotypes. The predicted capsids consistently exhibited low RMSD values, high GDT scores and robust TM alignment metrics. This high degree of structural fidelity underscores the reliability of the transformation matrices and SWISS-MODEL predictions utilised within CapBuild's workflow. However, care should be taken as assembly may not necessarily translate to function. As CapBuild performs capsid assembly through monomer matrix transformation, it is possible that the platform could assemble structures for mutants that are known to be defective in assembly. Therefore, downstream experimental validation is still critical.

CapBuild also integrates seamlessly with the broader ecosystem of molecular design and visualisation tools, such as Mol*, enabling researchers to analyse results interactively in three dimensions. The incorporation of customisable outputs, including PDB files annotated with beta values and CSV files detailing residue classifications, enhances its utility for both experimental and computational studies. These features render CapBuild not only a modelling tool but also a resource for hypothesis generation and experimental design.

In contrast to existing tools that may necessitate extensive computational expertise, CapBuild facilitates access to AAV engineering workflows, enabling researchers across disciplines to engage with capsid modelling and analysis. The web server’s integration of pre-computed transformation matrices, and rigorous geometric methodologies ensure that users can obtain the most accurate and current results.

Future enhancements to CapBuild could focus on incorporating additional functionalities to further expand its utility. These enhancements may include enabling the modelling and assembly of capsids for a broader range of viruses that exhibit symmetric arrangements, such as icosahedral or helical symmetry. Furthermore, features such as advanced mutational analysis, stability prediction under various conditions and integrations with molecular dynamics simulations could significantly enhance its capabilities. By broadening its scope and refining its user interface, CapBuild has the potential to not only remain a fundamental tool for AAV-based therapeutics but also to serve as a versatile platform for studying and engineering other symmetric viral capsids, thereby facilitating advancements across virology and therapeutic design.

## Data Availability

CapBuild is accessible at https://capbuild.csiro.au/.
